# m(6)A Modification of lncRNA NEAT1 Regulates Chronic Myelocytic Leukemia Progression *via* miR-766-5p/CDKN1A Axis

**DOI:** 10.3389/fonc.2021.679634

**Published:** 2021-07-20

**Authors:** Fang-Yi Yao, Cui Zhao, Fang-Min Zhong, Ting-Yu Qin, Fang Wen, Mei-Yong Li, Jing Liu, Bo Huang, Xiao-Zhong Wang

**Affiliations:** ^1^ Jiangxi Province Key Laboratory of Laboratory Medicine, Department of Clinical Laboratory, The Second Affiliated Hospital of Nanchang University, Nanchang, China; ^2^ Department of Clinical Laboratory, The First Affiliated Hospital of Nanchang University, Nanchang, China

**Keywords:** chronic myeloid leukemia, NEAT1, miR-766-5p, CDKN1A, N6-methyladenosine (m6A) modification, METTL3

## Abstract

**Background:**

Chronic myeloid leukemia (CML) is an acquired hematopoietic stem malignant disease originating from the myeloid system. Long non-coding RNAs (lncRNAs) have been widely explored in cancer tumorigenesis. However, their roles in CML remain largely unclear.

**Methods:**

The peripheral blood mononuclear cells (PBMCs) and CML cell lines (K562, KCL22, MEG01, BV173) were collected for *in vitro* research. Real-time quantitative polymerase chain reaction was used to determine the mRNA expression levels. Cell viability and apoptosis were analyzed by cell counting kit 8 and flow cytometry assays. The targeting relationships were predicted using Starbase and TargetScan and ulteriorly verified by RNA pull-down and luciferase reporter assays. Western blotting assay was performed to assess the protein expressions. N6-methyladenosine (m6A) modification sites were predicted by SRAMP and confirmed by Methylated RNA immunoprecipitation (MeRIP) assay.

**Results:**

LncRNA nuclear-enriched abundant transcript 1 (NEAT1) expression levels were decreased in the CML cell lines and PBMCs of CML patients. Moreover, METTL3-mediated m6A modification induced the aberrant expression of NEAT1 in CML. Overexpression of NEAT1 inhibited cell viability and promoted the apoptosis of CML cells. Additionally, miR-766-5p was upregulated in CML PBMCs and abrogated the effects of NEAT1 on cell viability and apoptosis of the CML cells. Further, CDKN1A was proved to be the target gene of miR-766-5p and was downregulated in the CML PBMCs. Knockdown of CDKN1A reversed the effects of NEAT1.

**Conclusion:**

The current research elucidates a novel METTL3/NEAT1/miR-766-5p/CDKN1A axis which plays a critical role in the progression of CML.

## Introduction

Chronic myelocytic leukemia (CML) is a malignant clonal proliferative disease that manifests with a large number of immature white blood cells accumulating in the bone marrow, which inhibits the normal hematopoiesis of the bone marrow (1). CML patients often suffer a characteristic reciprocal translocation t(9:22) (q34:q11) (Philadelphia chromosome) between chromosomes 9 and 22, forming a *bcr-abl* fusion gene, which aberrant expresses BCR-ABL protein with continuous tyrosine kinase activity (2). BCR-ABL activates downstream signal transduction pathways, thereby regulates the expression of cytokines, further leading to immature myeloid cells release into the peripheral blood, that is, occurrence of CML (3). Clinically, tyrosine kinase inhibitors (TKIs) are used to treat CML, but the effect of TKIs in relapsed CML patients is unsatisfactory (4). Therefore, to identify the novel therapeutic targets for CML is an urgent need.

Long non-coding RNAs (lncRNAs) and microRNAs (miRNAs) are both members of the non-coding RNA family (5). lncRNAs function as a ceRNA and regulate the development of various diseases by competitively binding to miRNAs (6). miRNAs further target the downstream genes (7). lncRNA nuclear enriched abundant transcript 1 (NEAT1) is an essential structural component of the subnuclear structure paraspeckle (8) and is closely related to malignant tumors and innate immunity (9). NEAT1 is demonstrated to be involved in acute promyelocytic leukemia (APL), acute myeloid leukemia (AML), acute lymphoblastic leukemia (ALL), and also CML (9–12). However, its role in CML is poorly understood.

The modified RNA plays a vital role in the post-transcriptional regulation of gene expression (13). In eukaryotes, N6-methyladenosine (m6A) modification is the most common form of RNA modification; its abundance has been found to account for 0.1–0.4% of the total adenosine residues (14). Generally speaking, m6A is highly conserved in humans and mice and regulates RNA stability, splicing, intracellular distribution, and translation (15). The cellular m6A state is mediated by a set of genes called “writers” (WTAP, METTL3, METTLI4), “erasers” (FTO, ALKBHS), and “readers” (YTHDF2, YTHDF2, YTHDF3, YTHDCI, YTHDC2). The writers form a multi-subunit methyltransferase complex, which upregulates m6A levels, while the erasers are m6A demethylase, which make the process reversible (16). A study proves that METTL3, a major RNA N6-adenosine methyltransferase, is identified as an essential gene for growth of AML cells (17). In addition, METTL3 promotes chemoresistance and attenuates autophagy of CML cells (18). Although the m6A modification of RNA has been found to be involved in the occurrence of different types of cancer, little is known about the relationship between m6A-related RNA and CML.

This study aimed to reveal the roles of NEAT1 in CML. In addition, m6A modification of NEAT1 was investigated.

## Material and Methods

### Patient Samples

The peripheral blood samples were collected in the First/Second Affiliated Hospital of Nanchang University from 2019 to 2020. The CML patients were pathologically diagnosed and grouped into CML-CP (n = 20) and CML-BC (n = 20). The control samples (n = 20) were obtained from healthy individuals. Peripheral blood mononuclear cells (PBMCs) were isolated by Ficoll-Histopaque (Sigma, USA). The enrolled donors did not undergo chemotherapy, radiotherapy, nor did they have organ dysfunction or pregnancy. The study was approved by the Ethics Committee of the First/Second Affiliated Hospital of Nanchang University and strictly observed the Declaration of Helsinki. All samples were anonymous, and signed informed consents were obtained from each individual.

### Cell Culture and Transfection

K562, KCL22, MEG01, and BV173 cell lines were purchased from Institute of Hematology, Chinese Academy of Medical Sciences. Cells were maintained in RPMI-1640 containing 10% FBS, 100 U/ml penicillin, and 100 mg/ml streptomycin (all from Gibco) at 37°C in the presence of 5% CO_2_. Transfection was carried out when the cell confluence reached 70–80%. pcDNA3.1/NEAT1, miR-766-5p mimic/inhibitor, CDKN1A small interference RNA (si-CDKN1A), si-METTL3, and their negative controls (Abiocenter Biotech Co., Ltd.) were transfected into the cells with Lipofectamine^®^ 2000 reagent (Invitrogen) according to the manufacturer’s protocol.

### Real-Time Quantitative Polymerase Chain Reaction

PBMCs and CML cells were mixed with TRIzol^®^ reagent (Invitrogen; Thermo Fisher Scientific, Inc.) to extract total RNA. Reverse transcription and qPCR were carried out using BlazeTaq One-Step SYBR Green RT-qPCR Kit (with ROX) (QP071; GeneCopoeia Inc.) on SEDI Thermo Cycler controlled by the Control Bus Net software package (Wealtec Bioscience Co., Ltd). All primers were designed and synthesized by Nanjing Genscript Biotech Co., Ltd.; GAPDH and U6 were used for internal reference. Fold changes of the indicated genes were calculated using 2^-ΔΔCt^ method. The sequences of the primers used were as follows: NEAT1: F, 5′-GTACGCGGGCAGACTAACAC-3′, R, 5′-TGCGTCTAGACACCACAACC-3′; miR-766-5p: F, 5′-TAAAATAGGAGTACTGTCTAA-3′, R, 5′-ATTAGTAAATTGGCTGCTGCAG-3ʹ; CDKN1A: F, 5′‐CAGCAGACCACCATTTCA-3′, R, 5′-GGTGTCTAGGTGCTCCAGGT-3′; METTL3: F, 5′-CAAGCTGCACTTCAGACGAA-3′, R, 5′-GCTTGGCGTGTGG TCTTT-3′; GAPDH: F, 5′-ATGGTGAAGGTCGGTGTGAA-3′, R, 5′-GAGTGGAGTCATACTGGAAC-3′; U6: F, 5′-GCTTCGGCAGCACATATACTAAAAT-3′, R, 5′-CGCTTCACGAATTTGCGTGTCAT-3′.

### Cell Counting Kit 8

The cells were resuspended at 1 × 10^5^ cells/ml then seeded into the 96-well plates at 100 μl/well. Then 10 μl of CCK8 reagent (AMJ-KT0001; AmyJet Technology Co., Ltd.) was added to each well of the plate and cultured in the incubator at 37°C for 4 h to detect cell proliferation. The absorbance values were evaluated with a microplate reader (HBS-1096C; Nanjing DeTie Experimental Equipment Co., Ltd.) at the wavelength of 490 nm.

### Flow Cytometry Assay

The cells were stained by TransDetect^®^ Annexin V-FITC/PI Cell Apoptosis Detection Kit (FA101-01; TransGen Biotech Co., Ltd.). 2 × 10^5^ cells were gathered; 5 μl of the Annexin V-FITC was added to each well of the six-well plate and incubated in the darkroom for 15 min under room temperature. NovoCyte Advanteon B4 Flow Cytometer and NovoSampler Q software (Agilent Technologies Co., Ltd.) were used for analysis.

### Luciferase Reporter Assay

The wild (WT) and mutant (MUT) type 3′-UTR regions of NEAT1 and CDKN1A luciferase reporter vectors were designed and synthesized by Guangzhou RiboBio Co., Ltd. The cells were incubated for 24  h. The cells were lysed to detect the luciferase activities using the Luciferase Reporter Assay Kit (K801-200; BioVision Tech Co., Ltd.) 48 h after co-transfection with the luciferase reporter vectors and miR-766-5p mimic/control. The luciferase activity was normalized to Renilla luciferase activity.

### RNA Pull-Down

RNA pull-down assay was performed by MagCapture™ RNA Pull-Down Assay Kit (297-77501; Whatman Co., Ltd.) following the manufacturer’s protocol. Briefly, the cells were lysed and incubated with the biotinylated miR-766-5p probe and its control probe. Streptavidin-labeled magnetic beads were resuspended and incubated with the probes (50 pmol) at 4°C overnight. Next, the beads were eluted from the RNA–protein complex. The proteins were then collected for mass spectrometry analysis.

### Western Blotting Assay

Total protein was extracted by pre-chilled RIPA lysis buffer (P0013C) for 30 min and quantified using a BCA protein Assay Kit (P0012S; both from Beyotime Institute of Biotechnology). Ten percent SDS-PAGE was used to separate the protein (40 μg) for 1.5 h at 120 V. Subsequently, the separated protein was transferred onto the PVDF membranes (Millipore) for 2 h at 200 mA. The membranes were then blocked with QuickBlock™ Blocking Buffer (Beyotime Institute of Biotechnology) for 1 h. Afterwards, the membranes were incubated with primary antibodies including anti-CDKN1A (1:1,000; PAB2922; Abnova Biotech Inc.) and anti-GAPDH (1:3,000; AMM04690G; Leading Biology) at 4°C on a shaking table overnight. GAPDH was used to normalize protein expression levels. Thereafter, the membranes were incubated with a horseradish peroxidase conjugated secondary antibody (1:1,000; 6916; BioVision) at room temperature for 2 h. Finally, protein bands were visualized using an ECL system (Thermo Fisher Scientific, Inc.).

### M6A Modification Site Prediction

The potential m6A modification sites of NEAT1 were predicted by SRAMP (http://www.cuilab.cn/sramp).

### Methylated RNA Immunoprecipitation Assay

Magna MeRIP™ m6A Kit (A-17-10499; Millipore) was used to conduct MeRIP according to the manufacturer’s protocol. Briefly, 30 μg of the total RNA was collected and mixed with the MeRIP reagent. Magna ChIP protein A/G Magnetic Beads were resuspended and conjugated to anti-m6A (MABE1006) or normal mouse anti-IgG antibodies (CS200621; both from A&D Technology Co., Ltd.) overnight at 4°C. Finally, the magnetic beads were eluted from the complex, and the RNA level was quantified using RT-qPCR as described above.

### Statistical Analysis

Each experiment was performed for at least three times. Data were analyzed by GraphPad Prism (version 7, GraphPad Software Inc.). The data were presented as mean ± SD. The student t-test was performed for the two-group comparison, and the analysis of variance (ANOVA) followed by Duncan’s *post-hoc* test was used for the comparison among multiple groups. P <0.05 suggested a significant difference.

## Results

### The Expression of NEAT1 Is Decreased in CML Patients and Cells

**Figure 1:**
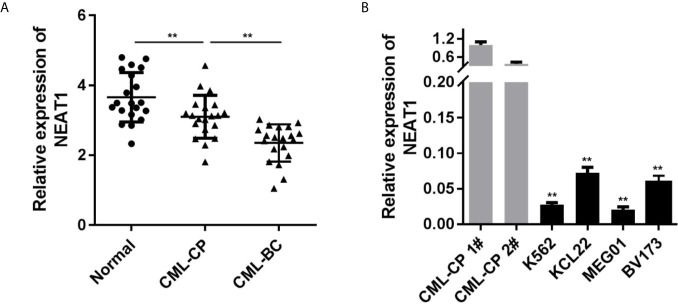
NEAT1 is downregulated in CML. **(A)** The expression of NEAT1 in CML patients. **(B)** The expression of NEAT1 in CML cell lines. CML, chronic myeloid leukemia; NEAT1, lncRNA nuclear enriched abundant transcript 1; CP, chronic phase; BC, blast crisis. ^**^P < 0.01 *vs.* normal or CML-CP.

Firstly, we evaluated the NEAT1 expression levels in clinical samples. Compared with the control group, the expression of NEAT1 in CML patients were notably decreased, which was more potent in the CML-BC group (**Figure 1A**). Moreover, NEAT1 expressions in four types of CML cell lines (K562, KCL22, MEG01, BV173) were all remarkably reduced (**Figure 1B**), which was more remarkable in K562 and KCL22 cells.

### NEAT1 Suppresses Cell Proliferation While Promotes Apoptosis of the K562 and KCL22 Cells

**Figure 2:**
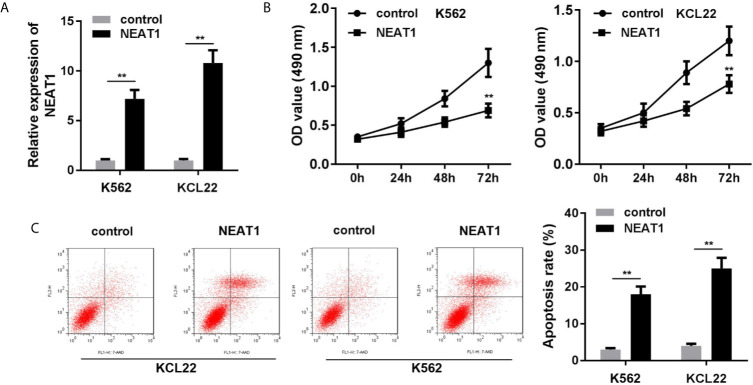
Overexpression of NEAT1 suppresses cell viability and inhibited the apoptosis in CML cells. **(A)** The expression of NEAT1 in CML cells. **(B)** Cell viability after incubation for 0, 24, 48, 72 h in the cells transfected with NEAT1/pcDNA3.1. **(C)** The apoptosis rates of CML cells. ^**^P < 0.01 *vs.* control. NEAT1, lncRNA nuclear enriched abundant transcript 1.

As shown in **Figure 2A**, the expression of NEAT1 was significantly increased, suggesting cells were successfully transfected. Overexpression of NEAT1 significantly repressed cell viability (**Figure 2B**) and accelerated apoptosis of K562 and KCL22 cells (**Figure 2C**).

### miR-766-5p Directly Binds With NEAT1 and Is Upregulated in CML Patients

**Figure 3:**
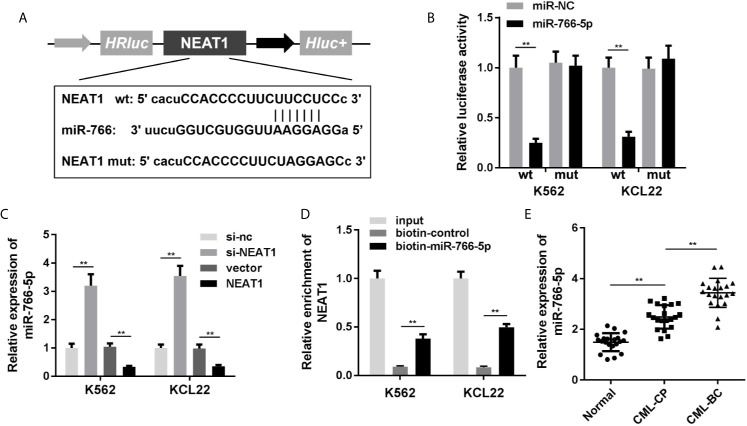
NEAT1 sponges miR-766-5p. **(A)** The binding sites between miR-766-5p NEAT1. **(B)** The luciferase activity of CML cells. **(C)** The expression of miR-766-5p in CML cells. **(D)** The interaction between NEAT1 and miR-766-5p. **(E)** The expression of miR-766-5p in CML patients. ^**^P < 0.01 *vs.* miR-NC, si-NC, vector, biotin-NC or normal. CML, chronic myeloid leukemia; NEAT1, lncRNA nuclear enriched abundant transcript 1; CP, chronic phase; BC, blast crisis; NC, negative control.

NEAT1 might function as ceRNA and modulate biological processes *via* sponging miRNAs as we described above. So, we used the online database Starbase 3.0 (http://starbase.sysu.edu.cn/) to predict the potential target miRNAs of NEAT1. The predictive analysis showed miR-766-5p was one of the targets of NEAT1, thus we transfected the luciferase reporters of NEAT1 into the cells to confirm the prediction (**Figure 3A**). The luciferase activity of the miR-766-5p and wild type NEAT1 co-transfection group was dramatically decreased (**Figure 3B**). Furthermore, the expression of miR-766-5p was significantly decreased by NEAT1 overexpression in the K562 and KCL22 cells, while it increased by NEAT1 knockdown (**Figure 3C**). In addition, biotinylated miR-766-5p group enriched notably higher level of NEAT1 (**Figure 3D**). Clinically, miR-766-5p was markedly increased in CML patients (**Figure 3E**).

### Overexpression of miR-766-5p Reverses the Effects of NEAT1 on Cell Viability and Apoptosis of the CML Cells

**Figure 4:**
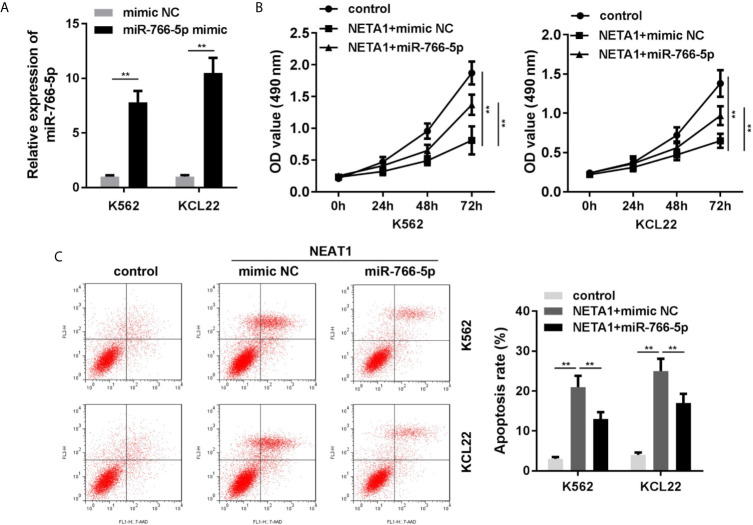
Overexpressed miR-766-5p enhances cell viability and inhibits the apoptosis of CML cells. **(A)** The transfection efficiency of miR-766-5p in CML cells. **(B)** The cell viability and **(C)** apoptosis of CML cells. ^**^P < 0.01 *vs.* mimic NC, control or NEAT1 + mimic NC. NEAT1, lncRNA nuclear enriched abundant transcript 1; NC, negative control.

Rescue assays were performed to verify the roles of NEAT1 and miR-766-5p in CML. As shown in **Figure 4A**, the expression of miR-766-5p was significantly increased in the miR-766-5p mimic group. Moreover, excess amount of miR-766-5p suppressed the effects of NEAT1 and promoted cell viability (**Figure 4B**) and restrained apoptosis (**Figure 4C**) of CML cells.

### CDKN1A Is Downregulated in the CML Cells as the Target Gene of miR-766-5p

**Figure 5:**
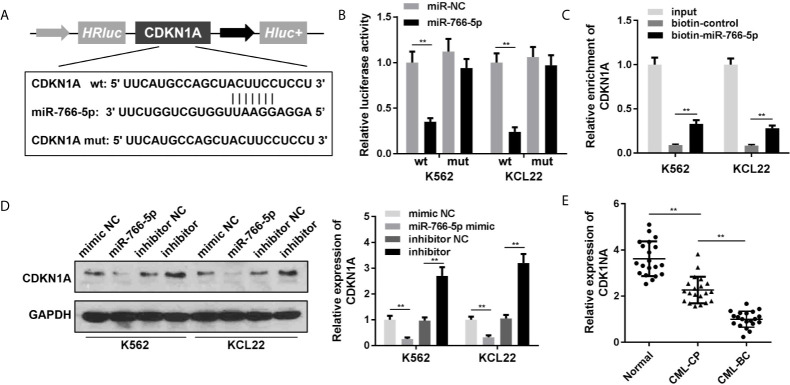
CDKN1A is a target of miR-766-5p. **(A)** The binding sites between miR-766-5p and CDKN1A. **(B)** The luciferase activities of CML cells. **(C)** The interaction between CDKN1A and miR-766-5p. **(D)** The protein expression of CDKN1A protein in CML cells. **(E)** The expression of CDKN1A in CML cells. ^**^P < 0.01 *vs.* miR-NC, si-NC, vector, biotin-NC or normal. CML, chronic myeloid leukemia; NEAT1, lncRNA nuclear enriched abundant transcript 1; CP, chronic phase; BC, blast crisis; NC, negative control.

To acquire the particular regulatory pathway consisted of NEAT1 and miR-766-5p, CDKN1A was predicted to be the target gene of miR-766-5p using TargetScan (http://www.targetscan.org/mamm_31/). Wild and mutant types of CDNK1A were synthesized and co-transfected with miR-766-5p into the cells (**Figure 5A**). Luciferase activity and RNA pull-down assay further verified the interaction between miR-766-5p and CDKN1A (**Figures 5B, C** **)**. In addition, miR-766-5p negatively regulated the protein expression of CDKN1A (**Figure 5D**). The mRNA expression of CDKN1A was significantly decreased in the CML patients (**Figure 5E**).

### Knockdown of CDKN1A Partially Abrogated the Effects of NEAT1

**Figure 6:**
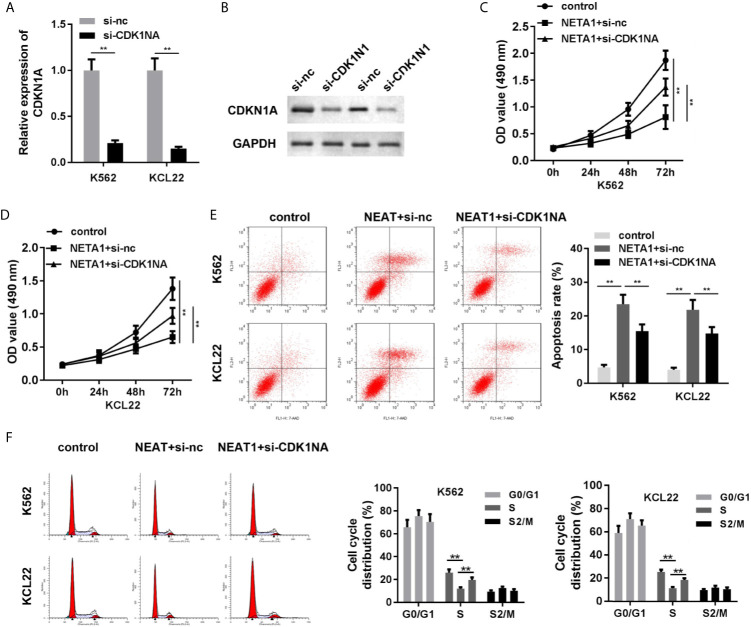
Knockdown of CDKN1A partially abrogates the effects of NEAT1 on cell viability and apoptosis. **(A, B)** The transfection efficiency of CDKN1A was evaluated by qPCR and western blot analysis. **(C, D)** The cell viability and **(E)** cell apoptosis of CML cells. **(F)** The cell cycle was evaluated by PI staining and flow cytometry. ^**^P < 0.01 *vs.* si-NC, control or NEAT1 + si-NC. NEAT1, lncRNA nuclear enriched abundant transcript 1; NC, negative control; CML, chronic myeloid leukemia; CDKN1A, cyclin-dependent kinase inhibitors 1A.

As shown in **Figures 6A, B**, the mRNA and protein expression of CDKN1A was significantly suppressed in si-CDKN1A group, indicating that cells were successfully transfected. Knockdown of CDKN1A promoted cell viability (**Figures 6C, D** **)** and inhibited apoptosis (**Figure 6E**) of CML cells. CDK1NA plays critical roles in the regulation of cell cycle. We further investigated the cell cycle of CML cells. The results indicated that NEAT1 overexpression induced S phase cell cycle arrest, while CDK1NA knockdown reversed this effect (**Figure 6F**).

### METTL3-Mediated m6A Modification Is Associated With the Downregulation of NEAT1 in CML

**Figure 7:**
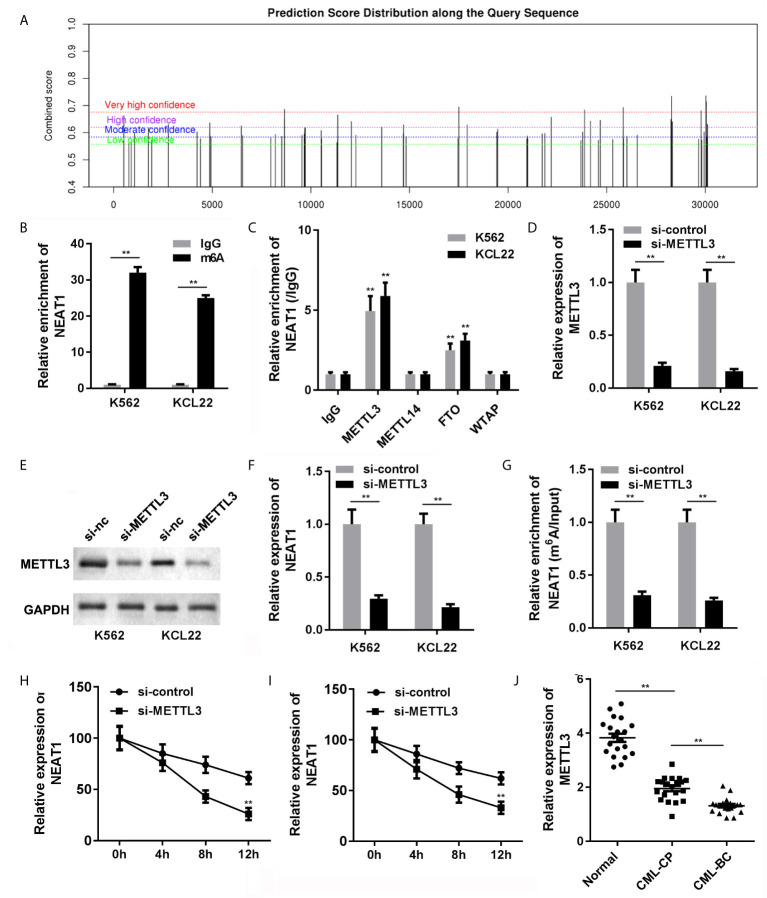
The causal of NEAT1 deficiency in CML. **(A)** The potential m6A modification sites of NEAT1 were predicted by SRAMP. **(B)** Anti-m6A antibodies enriched NEAT1 levels in the CML cells. **(C)** The interaction between NEAT1 and METTL3, METTL14, FTP, or WTAP. **(D)** The expression of METTL3 in CML cells. **(E)** The expression of NEAT1 in CML cells. **(F)** METTL3 induced m6A modification of NEAT1. **(G, H)** The mRNA stability of NEAT1 in CML cells. **(I)** The expression of METTL3 in CML patients. **(J)** The expression of METTL3 in CML cells. ^**^P < 0.01 *vs.* IgG or si-NC. CML, chronic myeloid leukemia; NEAT1, lncRNA nuclear enriched abundant transcript 1; CP, chronic phase; BC, blast crisis; NC, negative control; METTL3, methyltransferase like 3.

The NEAT1/miR-766-5p/CDKN1A regulatory axis was confirmed in CML, ulteriorly prompting us to seek the regulating mode of NEAT1. SRAMP predictive analysis result exhibited that m6A modification sites were abundant in NEAT1, indicating that NEAT1 was highly possible to be modified *via* m6A methylation (**Figure 7A**). Besides, the MeRIP result verified that NEAT1 was m6A modified due to the prominently higher level of NEAT1 in the m6A group (**Figure 7B**). Then, we tried to find out which m6A-realated enzyme was involved in the modification of NEAT1. RIP assay was performed, and we found that METTL3 and FTO could enrich NEAT1 (**Figure 7C**). Subsequently, we selected METTL3 for further studies. Si-METTL3 was synthesized and transfected into the K562 and KCL22 cells; it markedly attenuated the expression of METTL3 (**Figures 7D, E**) and NEAT1 (**Figure 7F**) expressions as well decreased the m6A modification of NEAT1 (**Figure 7G**). In addition, knockdown of METTL3 accelerated the degradation of NEAT1 (**Figures 7H, I** **)**. What’s more, METTL3 levels of the CML patients in both CP and BC stages were significantly decreased (**Figure 7J**).

### NEAT1 Inhibited the Growth of CML Cells *In Vivo*

**Figure 8:**
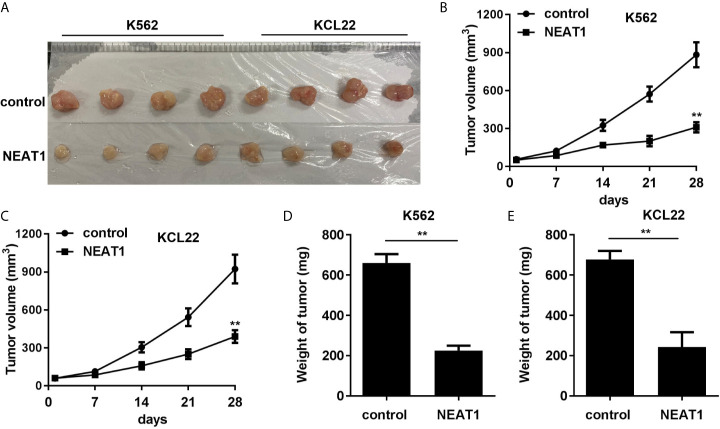
NEAT1 inhibited the growth of CML cells *in vivo*. **(A)** The image of tumor in control and NEAT1 group. **(B, C)** The volume of the tumors was assessed. **(D, E)** The weight of the tumors was detected. ^**^P < 0.01 *vs.* control group. NEAT1, lncRNA nuclear enriched abundant transcript 1; NC, negative control; CML, chronic myeloid leukemia; CDKN1A.

Animal studies were further carried out to evaluate the effect of NEAT1 on CML progression. We found that NEAT1 overexpression significantly reduced the volume and weight of the CML tumors (**Figures 8A–E**).

## Discussion

CML is a hematopoietic stem cell clonal disorder, and it accounts for approximately 15–20% of newly diagnosed leukemia in adults (19). LncRNAs have been revealed to participate in the progressions of CML through acting as ceRNA for miRNAs, and miRNAs further targeting for the abnormally expressed genes in CML (20). However, few research studies focus on the upstream regulation of lncRNAs. Recently, m6A modification has become an epigenetic hot point (11). Therefore, in the current study, we explored the regulatory axis of NEAT1 in CML and ulteriorly investigated the upstream modification of NEAT1. The data suggested that NEAT1 was inhibited in PBMCs of the CML patients and CML cell lines. NEAT1 overexpression ameliorated aggressiveness of the CML cells *via* the miR-766-5p/CDKN1A axis. Furthermore, NEAT1 received upstream m6A modification of METTL3. Knockdown of METTL3 accelerated the degradation of NEAT1 in the CML cells.

Numerous studies have demonstrated that dysregulation of NEAT1 is closely related to the progression of hematological malignancies (21). NEAT1 is downregulated in leukemia patients and cells (9). For example, NEAT1 was inhibited in AML. Overexpression of NEAT1 suppressed cell proliferation, migration, and invasion, meanwhile induced apoptosis of AML cells (22). Furthermore, NEAT1 exhibited therapeutic function on AML through affecting the cell cycle of the AML cells (23). Herein, we proved that NEAT1 was notably downregulated in CML patient and CML cells, which was in line with the previous studies (24). Overexpressed NEAT1 in K562 and KCL22 cells aggravated cell apoptosis and inhibited cell viability of CML cells, implying NEAT1 as a potential therapeutic target for CML.

As NEAT1 was downregulated in CML, we subsequently attempted to explore the molecular mechanism that led to NEAT1 downregulation. Emerging lines of evidence indicate that RNA methylation is a key factor for dysregulated lncRNAs, such as XIST (25), NEAT1 (26), MALAT1 (27), and lincRNA1281 (28). m6A modification is the most common form of RNA modification (29). We predicted whether there is m6A modification site in the sequence of NEAT1. Positively, we found several sites of very high confidence. Therefore, we hypothesized that the abnormal levels of NEAT1 in CML may be associated with m6A modification. The RNA pull-down assay confirmed our hypothesis. NEAT1 can bind with METTL3 and FTO. METTL3, a “writer” of m6A, was downregulated in CML. Silencing METTL3 lowered the m6A methylation and expression levels of NEAT1. METTL3 is one of the RNA methyltransferases, which participate in the regulation of mRNA stability, degradation, and translation through m6A modification. In this study, knockdown of METTL3 suppressed the expression mRNA stability of NEAT1. Therefore, the deficiency of METTL3 loses its ability to m6A modification of NEAT1.

To further obtain the detailed regulatory mechanism of NEAT1 on CML, we carried out predictive analysis, and miR-766-5p was predicted to be the target miRNA of NEAT1. miR-766-5p was first identified to be overexpressed in the colorectal cancer (CRC) tissue. Overexpression of miR-766-5p promotes the cell invasion and proliferation of CRC cells (30). In addition, inhibition of miR-766-5p relieves the progression of cervical cancer (31). Nevertheless, miR-766-5p is barely investigated in CML. In this study, miR-766-5p was overexpressed in CML patients. Increasing evidence reveals that lncRNAs function as a ceRNA to regulate the expression of miRNAs at post-transcriptional level (32). In this study, NEAT1 functioned as a sponge for miR-766-5p. Overexpressed miR-766-5p partially abrogated the effects of NEAT1 and modulated the proliferation and apoptosis of CML cells. Therefore, NEAT1 may participate in the progression of CML *via* suppressing miR-766-5p.

Next, we used the bioinformatic approaches to predict the target gene of miR-766-5p to further investigate the regulatory axis. Cyclin-dependent kinase inhibitor 1A (CDKN1A) is a member of the cyclin-dependent kinase inhibitor (CDKI) family, serving as a negative cell cycle regulator (33). A study reveals that overexpression of CDKN1A mediates cell cycle arrest at the G0/G1 phase in CML (34). CDKN1A may dominate the drug responsiveness of acute lymphoblastic leukemia (ALL) cells. CDKN1A positive ALL cells confer high sensitivity to Aurora kinase inhibitors, which promised CDKN1A as a potential biomarker in assessing the drug responsiveness of Aurora kinase inhibitors in ALL (35). In this study, CDKN1A was predicted and proved to be the target gene of miR-766-5p in CML. Knockdown of CDKN1A abrogated the effects of NEAT1 and promoted the aggressiveness of CML cells.

Taken together, NEAT1 was downregulated in CML, which was due to the deficiency of METTL3. Overexpressed NEAT1 suppressed the progression of CML *via* regulating miR-766-5p/CDKN1A axis. These findings may provide potential diagnostic indicator or therapeutical target for CML. However, much more research work should be carried out for the clinical use of it.

## Data Availability Statement

The datasets presented in this study can be found in online repositories. The names of the repository/repositories and accession number(s) can be found in the article/supplementary material.

## Author Contributions

All authors contributed equally. All authors contributed to the article and approved the submitted version.

## Funding

This work was supported by the National Natural Science Foundation of China (Grant No. 81360083, No. 81271912, No. 81560033 and No. 81860034).

## Conflict of Interest

The authors declare that the research was conducted in the absence of any commercial or financial relationships that could be construed as a potential conflict of interest.
